# Analysis of Dermatology Content by Top Influencers on Twitter and Their Academic Impact: Cross-Sectional Study

**DOI:** 10.2196/34742

**Published:** 2023-07-18

**Authors:** Mindy D Szeto, Andrina V Mamo, Kevin Kamel, Jadesola T Olayinka, Payal M Patel, Austin Hamp, Jarett Anderson, Lori S Kim, Madeleine G Yemc, Torunn E Sivesind, Robert P Dellavalle

**Affiliations:** 1 Department of Dermatology University of Colorado Anschutz Medical Campus Aurora, CO United States; 2 School of Medicine University of Colorado Anschutz Medical Campus Aurora, CO United States; 3 Department of Dermatology College of Medicine Downstate Health Sciences University Brooklyn, NY United States; 4 Department of Dermatology Massachusetts General Hospital Harvard University Boston, MA United States; 5 Department of Dermatology Beaumont Health Detroit, MI United States; 6 Department of Dermatology Boston University Boston, MA United States; 7 Dermatology Service Rocky Mountain Regional VA Medical Center Aurora, CO United States

**Keywords:** dermatology, social media, Twitter, influencers, publication citations, h-index, board certified, board certification, education

## Introduction

Social media platforms such as Twitter allow dermatologists to collaborate, share information with the public, expand their practices, and communicate directly with patients [1]. Concerningly, popular dermatology social media content is often generated by prolific content creators known as “influencers,” not board-certified dermatologists or individuals with evidence-based training [2]. We, therefore, conducted an analysis of top dermatology accounts on Twitter, the content posted by these accounts, and their users’ academic productivity and impact.

## Methods

The content aggregator Cronycle [3], which has been used in multiple peer-reviewed studies of social media in health care, was used to identify the top 100 Twitter accounts in May 2021 for the topic “dermatology.” Two independent researchers with experience in dermatology and social media extracted the accounts’ self-reported names, credentials, and geographic locations. Content from the 3 most recent Twitter posts for each account was categorized as “educational” if relaying dermatology or health care information, “professional” if associated with promoting conferences or professional events, “advertising” if related to specific products or services, or “personal” for all other posts. A consensus meeting resolved any discrepancies. Foreign language content was translated using Google Translate (Google LLC) if an English translation was not directly available from Twitter. Account users’ names were checked for American Board of Medical Specialties certifications through the Certification Matters search tool [4]. Academic citation metrics as of August 2021 (including the h-index [5] and the number of total publications and citations over an individual’s career) according to the Web of Science and Google Scholar databases were also recorded.

## Results

Of the top 100 accounts, 92 appeared to represent individuals and 8 were organizations (Table 1). Most were US board-certified physicians (n=52), and of these, 45 (87%) were dermatologists. However, credential verification was difficult for physicians certified or practicing outside the United States.

**Table 1:** Dermatology-related top influencers on Twitter in May 2021, number of followers, and board certification status or location outside of the United States.

Influencer rank	Twitter account	Name	Twitter followers, n	US board certified or non-US location
1	jamaderm	JAMA Dermatology	19,800	N/A^a^ (organization)
2	poschchristian	Christian Posch M.D. Ph.D.	16,900	Germany
3	ducrest	Dominique du Crest	8200	France
4	drdorisday	Dr Doris Day	9500	Yes; dermatology
5	dermatologistmd	Susan J. Huang MD	8600	Yes; dermatology
6	jmgardnermd	Jerad Gardner, MD	28,100	Yes; pathology, dermatopathology
7	drrodrohrich	Rod Rohrich, M.D.	133,900	Yes; plastic surgery
8	cdndermatology	Canadian Dermatology	5200	N/A (organization)
9	globaldermie	Josette McMichael MD FAAD	4500	Yes; dermatology
10	sergiovanog	Dr. Sergio Vañó	34,200	Spain
11	dermdoc	Jeff Benabio, MD MBA	22,000	Yes; dermatology
12	harveylui	Harvey Lui	2100	Yes; dermatology
13	socinvestderm	SID	2900	N/A (organization)
14	drstefaniew	Dr Stefanie Williams	12,200	United Kingdom
15	dranjalimahto	Dr Anjali Mahto	6300	United Kingdom
16	adeadamson	Ade Adamson, MD MPP	6100	Yes; dermatology
17	aedv_es	Academia Española de Dermatología y Venereología	9900	N/A (organization)
18	seemalrdesaimd	Seemal R. Desai, MD, FAAD	1900	Yes; dermatology
19	dermdrhale	Dr. Elizabeth Hale	3900	Yes; dermatology
20	carriekovarik	Carrie Kovarik, MD, FAAD	1500	Yes; dermatology, dermatopathology
21	roxanadaneshjou	Roxana Daneshjou MD/PhD	13,400	Yes; dermatology
22	juleslipoff	Jules Lipoff, MD	2900	Yes; dermatology
23	misharosenbach	Misha Rosenbach, MD	3300	Yes; dermatology
24	skinpathology	Paul Drury	6200	Australia
25	lupodermatology	Dr. Mary Lupo	3200	Yes; dermatology
26	drsteventchen	Steven Chen 陳持威	9300	Yes; dermatology, internal medicine
27	sgottesmanmd	Silvija Gottesman MD	6800	Yes; dermatology, dermatopathology
28	desaze	David Saceda Corralo	4300	Spain
29	drlopezbran	Eduardo López Bran	7600	Spain
30	antoniotejera	Antonio Tejera Vaquerizo	2500	Spain
31	dedeemurrell	Dedee Murrell	1400	Yes; dermatology
32	harkerdavid	David Harker, MD, FAAD	13,700	Yes; dermatology
33	dermatologaroo	Elia Roo	3000	Spain
34	drjohnbarbieri	John Barbieri, MD, MBA	2700	Yes; dermatology
35	drjoelgelfand	Joel M Gelfand MD MSCE FAAD	1800	Yes; dermatology
36	dravasays	Dr. Ava Shamban	10,000	Yes; dermatology
37	julianconejomir	Julián Conejo-Mir	8600	Spain
38	rosataberner	Rosa Taberner	17,000	Spain
39	dra_njimenez	Natalia Jiménez-Dermatóloga	4900	Spain
40	marcelasaebl	Marcela Saeb Lima	11,000	Mexico
41	elena_heras	Maria Elena de las Heras Alonso	2100	Spain
42	drboixeda	Pablo boixeda	2600	Spain
43	ealtmanmd	Emily M. Altman, MD	1600	Yes; dermatology, dermatopathology
44	doctormartorell	Dr.Antonio Martorell	2400	Spain
45	danielbutlermd	Daniel Butler MD	1500	Yes; dermatology
46	amostaghimi	Arash Mostaghimi	2100	Yes; dermatology, clinical informatics
47	hpsoyer	H. Peter Soyer	1900	Australia
48	lauzurica_derma	Eduardo Lauzurica	2700	Spain
49	dranthonyrossi	Dr. Anthony Rossi MD	1100	Yes; dermatology, micrographic dermatologic surgery
50	dermalegre	Adrián Alegre	2700	Spain
51	pielsana_aedv	Fundación Piel Sana de la AEDV	4800	N/A (organization)
52	drdidacbarco	Dr. Dídac Barco	3100	Spain
53	drramongrimalt	Dr. Ramon Grimalt	7500	Spain
54	drestherfreeman	Esther Freeman MD PhD	2100	Yes; dermatology
55	askdermmd	A. Shadi Kourosh, MD, MPH	11,500	Yes; dermatology
56	harrisvitiligo	John E. Harris, MD, PhD	3200	Yes; dermatology
57	drrobertanolik	Dr. Robert Anolik	2700	Yes; dermatology
58	msldermatopato	Marcel Saeb Lima	2800	Mexico
59	mightydermpath	Sara Shalin	3800	Yes; pathology, dermatopathology
60	dermatosilva	DOMINGUEZ SILVA	2600	Spain
61	drahermosag	Ángela Hermosa	3200	Spain
62	lpdermatologos	Lidia Perez dermatologos	1200	Spain
63	dermoguillenvlc	Carlos Guillen	1600	Spain
64	allisonlarsonmd	Allison Larson, MD	2100	Yes; dermatology, dermatopathology
65	melanoma_mama	Donna Regen	2500	No; melanoma awareness activist
66	hlgreenberg	H.L. Greenberg, M.D.	1700	Yes; dermatology
67	mrodriguesmd	Michelle Rodrigues	1300	Australia
68	katiefarquhar	Dr Katie Farquhar	1400	United Kingdom
69	dolevderm	Jacqueline Dolev MD	1500	Yes; dermatology
70	franvilmar	Francisco Vílchez	1700	Spain
71	larocheposayusa	La Roche-Posay USA	8000	N/A (organization)
72	leohealthyskin	LEO Pharma	16,100	N/A (organization)
73	cbaileymd	Cynthia Bailey	4300	Yes; dermatology
74	drmichellelevy	Michelle Levy, MD, Dermatologist	1200	Yes; dermatology
75	drwhitneybowe	Dr. Whitney Bowe	4600	Yes; dermatology
76	aaron_drucker	Aaron Drucker	933	Yes; dermatology
77	condetaboada	Alberto Conde	1400	Spain
78	your_skin_dr	Dr Anton Alexandroff	4800	United Kingdom
79	beerdermatology	Beer Dermatology	2500	Yes; dermatology, dermatopathology
80	drgrantstevens	Grant Stevens, MD	5600	Yes; plastic surgery
81	springerderma	Springer Dermatology	1800	N/A (organization)
82	laumiguelg	Laura M.G.	1500	Spain
83	dermaforyou	Dra Carmen Galera	1500	Spain
84	ecoalfageme	Dr.Fernando Alfageme	1400	Spain
85	germainderm	Germain Dermatology	2500	Yes; dermatology
86	leoshmu	Leo Shmuylovich, MD PhD	1100	Yes; dermatology, pediatric dermatology
87	ilanarosman	Ilana Rosman, MD	3000	Yes; dermatology, dermatopathology
88	drkatiebeleznay	Dr. Katie Beleznay	1800	Yes; dermatology
89	doctoraston	Dr. Sherrell Aston	5100	Yes; surgery, plastic surgery
90	theskinmd	Jessica Krant MD	1700	Yes; dermatology
91	hellmanderm	Dr Judith Hellman	2100	Yes; dermatology
92	alvaro_gonza	Álvaro González-Cantero	1100	Spain
93	paolijohn	John Paoli	917	Sweden
94	elenacondemon	Elena Conde Montero	2300	Spain
95	mydermpath	Raj Singh MD	5100	Yes; pathology, dermatopathology
96	drbilumartin	Donna Bilu Martin, MD	2100	Yes; dermatology
97	dr_weiss	Robert A. Weiss	1100	Yes; dermatology
98	drdayan	Dr. Steven Dayan	2700	Yes; otolaryngology
99	_dermatologist	Dr Edward Seaton	3000	United Kingdom
100	drlisakates	Lisa Kates, M.D.	2000	Yes; dermatology

^a^N/A: not applicable.

**Figure 1:**
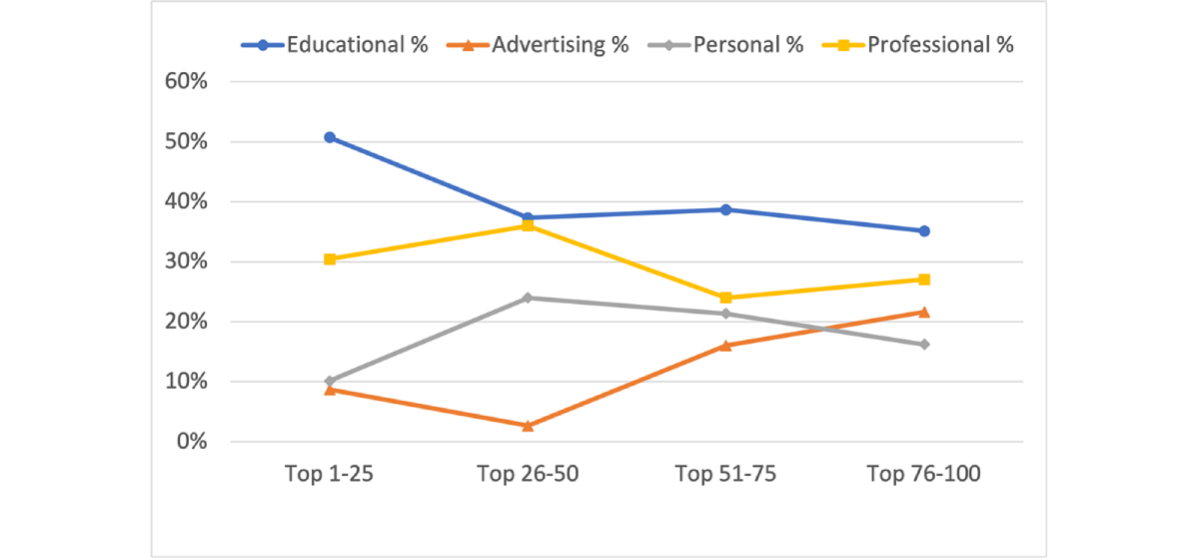
Percentages of educational, advertising, personal, and professional posts by dermatology-related top influencers on Twitter in May 2021.

Academic citation metrics according to the h-index (mean 14, SD 13; median 12, range 0-71) and total publications (mean 89, SD 148; median 43, range 2-950) were highly variable for 81 individuals with citation profiles. Educational posts were the most common (118/293, 40%), followed by professional content (86/293, 29%), personal posts (53/293, 18%), and lastly, advertising (36/293, 12%). Top quartile accounts by Cronycle rank posted far more educational content (>50%) than other categories of posts (Figure 1), and the educational category occurred most frequently in each quartile. Advertising was the least common content category, in contrast to other platforms such as Instagram, where the most common content was related to advertising [6] and posted predominantly by nondermatologist influencers [7].

## Discussion

We offer a cross-sectional snapshot of associations between content, popularity, and academics, prompting many avenues for further work and repeat assessment. For example, it remains to be seen whether social media branding affects academic influence, or vice versa. Twitter excels at encouraging user interactions through “mentions” and “follows,” with the “hashtag” function allowing easy searching and content propagation. However, without further protections, Twitter could also risk spreading harmful misinformation as an educationally oriented platform. While Twitter verification policies are currently in flux, account registration does not require identity verification, though this could be explored in the future to highlight board-certified physician accounts, especially for international dermatologists where external credential verification resources are limited.
